# Long-Term Survival of More than 5 Years with Maintenance Therapy Using Single-Agent Pemetrexed in a Patient with Diffuse Malignant Peritoneal Mesothelioma

**DOI:** 10.1155/2023/2092157

**Published:** 2023-02-28

**Authors:** Yasuo Otsuka, Yoriaki Komeda, Masayuki Takeda, Takayuki Takahama, Masashi Kono, Mamoru Takenaka, Satoru Hagiwara, Naoshi Nishida, Hiroshi Kashida, Masatoshi Kudo

**Affiliations:** ^1^Department of Gastroenterology and Hepatology, Kindai University Faculty of Medicine, Osaka-Sayama, Japan; ^2^Department of Cancer Genomics and Medical Oncology, Nara Medical University, 840 Shijo-Cho, Kashihara, Nara, Japan; ^3^Department of Medical Oncology, Kindai University Faculty of Medicine, Osaka-Sayama, Japan

## Abstract

A 76-year-old woman presented with lower abdominal pain and nausea and was referred to the gastroenterology department in our institution. Previous contrast-enhanced computed tomography (CE-CT) for follow-up after breast cancer surgery had indicated a soft tissue mass below the right diaphragm, which was considered a benign change. CE-CT performed at the first visit to our department revealed further thickening of the soft tissue mass with extension to the liver surface. In addition, ascites and nodules were observed in the abdominal cavity. Histopathological examination of a biopsy specimen revealed peritoneal invasion of atypical epithelioid cells with trabecular and glandular patterns. The tumor cells were positive for AE1/AE2, calretinin, WT-1, D2-40, HEG1, EMA, BAP1, and MTAP and negative for carcinoembryonic antigen, MOC-31, Ber-Ep4, ER, PgR, TTF-1, claudin 4, and desmin. A diagnosis of epithelioid mesothelioma was made. The patient received chemotherapy with cisplatin (75 mg/m^2^) and pemetrexed (500 mg/m^2^). After six courses of combined chemotherapy, pemetrexed was administered as a single agent. At the time of writing this report, she was undergoing over the 30th course of chemotherapy without any significant side effects. Diffuse malignant peritoneal mesothelioma is a rare, fatal, and progressive disease. Our patient achieved long-term survival of more than 5 years with maintenance therapy using single-agent pemetrexed.

## 1. Introduction

Malignant mesothelioma is a rare, insidious, aggressive tumor that originates from the mesothelial surface of the pleural and peritoneal cavities, the pericardium, or the tunica vaginalis. In the United States, malignant peritoneal mesothelioma (MPM) accounts for up to 15% of all cases of mesothelioma, and it is rapidly fatal. MPM is difficult to diagnose because of its nonspecific clinical symptoms and findings. The common symptoms are abdominal pain or a palpable abdominal mass. MPM often develops near the pelvis, rectum, urinary bladder, or uterus. Generally, it is treated with surgical resection or chemotherapy [1]. MPM is often located near the pelvis, rectum, urinary bladder, or uterus. Generally, the treatment method is surgical resection or chemotherapy. There is no effective treatment, and the median survival time ranges from 5 to 12 months [1]. Here, we report a case of an elderly woman with diffuse MPM who achieved long-term survival after six courses of combined chemotherapy with cisplatin (75 mg/m^2^) and pemetrexed (500 mg/m^2^), followed by maintenance therapy with single-agent pemetrexed.

## 2. Case Presentation

A 76-year-old woman was undergoing regular follow-ups at the Department of Breast Surgery in our hospital for postoperative breast cancer. There was no recurrence of breast cancer; however, she presented with a complaint of lower abdominal pain and nausea and was referred to the gastroenterology department. Her medical history did not include any apparent episodes of asbestos exposure. Physical examination at the first visit showed normal vital signs, a flat and soft abdomen, and tenderness in the lower abdomen.

**Figure 1:**
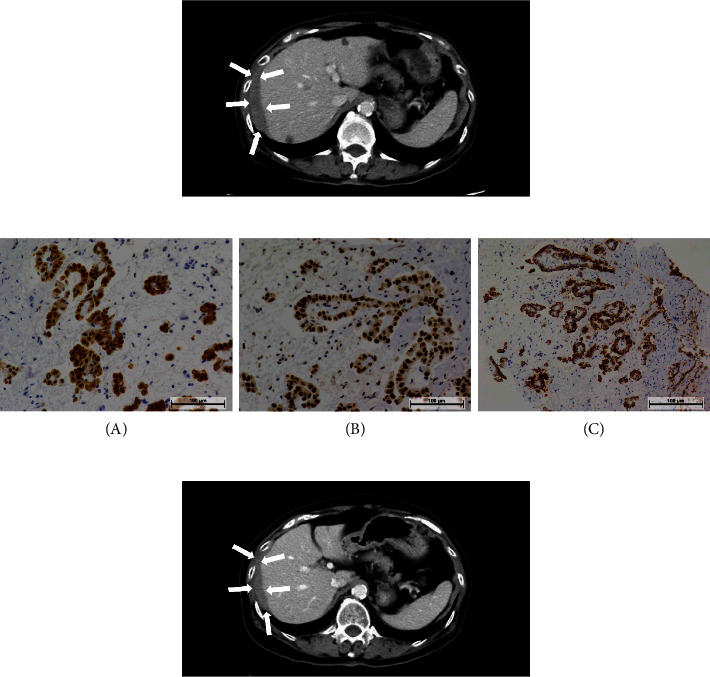
Imaging and immunohistochemistry of the lesion. (a) CE-CT image at the first visit to our department. The soft tissue mass is visible below the right diaphragm (arrows). (b) Immunohistochemical analysis of the lesion revealed that the tumor cells were positive for calretinin (A), WT-1 (B), and D2-40 (C). (c) CE-CT image at 48 months after the start of chemotherapy. The lesions were indicative of stable disease (arrows). CE-CT: contrast-enhanced computed tomography.

Blood tests showed no abnormal values, and tumor markers such as carcinoembryonic antigen (CEA), cancer antigen (CA) 19-9, and CA 125 were within normal limits. Previous contrast-enhanced CT (CE-CT) for follow-up after breast cancer surgery had indicated a soft tissue mass below the right diaphragm, which was considered a benign change. CE-CT performed at the first visit to our department revealed further thickening of the soft tissue mass with extension to the liver surface. In addition, ascites and nodules were observed in the abdominal cavity (Figure 1(a)). Fluorodeoxyglucose positron emission tomography (FDG-PET) revealed abnormal uptake by the soft tissue mass and nodules in the abdominal cavity. No abnormal uptake was observed in the other organs. On the basis of the FDG-PET findings, malignancy was suspected. A biopsy was performed under transabdominal wall ultrasound guidance, and histopathological examination revealed peritoneal invasion of atypical epithelioid cells with trabecular and glandular patterns. Atypical cells also invaded the skeletal muscles. Considering the skeletal muscle invasion, the atypical cells were suspected to be malignant epithelioid neoplasia. Immunohistochemistry revealed that the tumor cells were positive for AE1/AE2, calretinin, WT-1, D2-40, HEG1, EMA, BAP1, and MTAP and negative for CEA, MOC-31, Ber-Ep4, ER, PgR, TTF-1, claudin 4, and desmin. Based on the histopathological and immunohistochemical findings, the tumor was diagnosed as an epithelioid mesothelioma (Figure 1(b)-(A) = calretinin, (B) = WT-1, and (C) = D2-40). Because the FDG-PET findings strongly suggested peritoneal dissemination, radical treatment by surgical resection was considered difficult, and systemic chemotherapy was administered. Chemotherapy with cisplatin (75 mg/m^2^) and pemetrexed (500 mg/m^2^) was initiated. CT evaluation 12 months after the start of treatment showed no changes in the tumor, and the treatment was continued. After six courses of combined chemotherapy, pemetrexed was administered as a single agent because the total cisplatin dose exceeded 500 mg. At the time of writing this report, she was undergoing over the 30th course of chemotherapy without any significant side effects. CT performed 48 months after the start of chemotherapy showed stable disease with amelioration of the abdominal symptoms observed at the initial examination (Figure 1(c)).

## 3. Discussion

Peritoneal mesothelioma was first reported by Miller and Wynn in 1908. Among all mesotheliomas, peritoneal lesions are rare; most mesotheliomas occur in the pleura and peritoneum, with 80% involving the pleura and 20% involving the peritoneum [1]. Diffuse MPM, like the one observed in the present case, reportedly accounts for 10%–30% of all mesotheliomas [2]. Asbestos exposure is considered a major cause of pleural mesothelioma. Although the frequency of a history of asbestos exposure is lower for peritoneal mesothelioma than for pleural mesothelioma, an association with asbestos exposure has been suggested [1]. According to some reports on the prognosis of MPM, the 5-year survival rate is 29%–59%, and there are no specific symptoms or clinical features [1, 2]. MPM is categorized into three pathological subtypes: epithelioid (the most frequent), sarcomatoid, and mixed (biphasic) [3].

Measurement of biomarkers is recommended for the diagnosis of mesothelioma. In addition, the absence of BAP1 and MTAP loss is effective in distinguishing the epithelioid and sarcomatoid types; however, it is not 100% accurate, and peritoneal mesothelioma can also show the absence of MTAP loss. The treatment of mesothelioma includes chemotherapy and surgical resection. In addition, various treatment modalities are being explored, including radiation therapy and HIPEC [4]. One study suggests the possibility of radical surgical resection after HIPEC, but the rate of achievement is not high [5]. In this case, chemotherapy was chosen because of the possibility of peritoneal dissemination. In general, there is no established chemotherapy regimen for unresectable, advanced-stage peritoneal mesothelioma; regimens used for pleural mesothelioma are used for such cases. Pemetrexed in combination with cisplatin, carboplatin, or gemcitabine is recommended, and the combination of pemetrexed and cisplatin is widely used [4]. A phase III study of pemetrexed in combination with cisplatin for patients with MPM reported a response rate of 41.3% and a median survival time of 12.1 months [5]. Jänne et al. reported a response rate and median survival time of 23.3% and 13.1 months, respectively, for the same regimen in patients with peritoneal mesothelioma [6].

Targeted therapy, such as gene therapy, is based on the method of administering a thymidine kinase gene conjugate to the Herpes simplex virus and then administering an antiviral agent to kill it. This option could not be explored in the present case because of insufficient tissue mass in the peritoneal biopsy. Moreover, previous studies have evaluated the predicted biomarkers for pemetrexed-treated mesothelioma. High folylpoly-*γ*-glutamate synthetase and low thymidylate synthase levels have been associated with the sensitivity of pemetrexed; however, we did not examine these biomarkers in the present case. Given that comprehensive gene panel tests for advanced solid tumors are covered by insurance in Japan, it is possible to test these biomarkers if there is sufficient tumor volume.

In the present case, the patient was treated with chemotherapy comprising cisplatin (75 mg/m^2^) and pemetrexed (500 mg/m^2^), and cisplatin administration was interrupted because irreversible side effects such as peripheral neuropathy and renal failure occurred when the total dose exceeded 500 mg. Single-agent pemetrexed was administered after six courses of combined chemotherapy. For elderly and unfit patients in particular, single-agent pemetrexed (500 mg/m^2^ intravenously every 3 weeks) has been recommended [7]. Accordingly, maintenance chemotherapy with single-agent pemetrexed was selected for our patient, and she achieved long-term survival (>5 years) with this treatment; at the time of writing this report, she was still receiving chemotherapy without severe adverse events. It is considered that the long-term effect of the treatment led to prolonged survival. In the CALGB 30901 trial, maintenance therapy with pemetrexed following initial pemetrexed and platinum chemotherapy did not improve progression-free survival in patients with MPM [8]. Of late, cases of resectable MPM have been receiving combination therapy with cytoreductive surgery, such as total peritoneal resection and hyperthermic intraperitoneal chemotherapy [3]. Intraperitoneal administration of cytotoxic agents can improve their therapeutic activity. Determining the effect of systemic or intraperitoneal chemotherapy on tumor progression in these patients is difficult because of the lack of evidence in the medical article; therefore, further prospective studies are warranted. Peritoneal mesothelioma is a rare malignancy, and it is difficult to conduct large-scale clinical trials of patients with this condition. Since we report only one case, the utility of maintenance therapy with pemetrexed should be examined in a clinical trial. In addition, biomarker research on the long-term effects of this treatment should be conducted in the future.

In conclusion, diffuse MPM is a rare, fatal, and progressive disease. However, our patient achieved long-term survival of more than 5 years with maintenance therapy using single-agent pemetrexed. This may be because pemetrexed has no tolerable dose and is administered only once every three weeks with an infusion time of 30 minutes, ensuring good compliance and reducing patient burden. All these indicate that pemetrexed therapy may help in maintaining long-term tumor shrinkage without severe side effects.

## Data Availability

The data underlying the results presented in the study are available.
